# Analysis of a cohort of patients with indication of tracheostomy in intensive care medicine

**DOI:** 10.1186/2197-425X-3-S1-A939

**Published:** 2015-10-01

**Authors:** E Martínez Barrio, A Berrazueta Sánchez de Vega, J Romero Pellejero, M Del Valle Ortiz, JA Fernández Ratero, C Diaz Tobajas, D Armesto Formoso, E Portugal Rodríguez, C Carbajales Pérez, S Puerto Corrales

**Affiliations:** Burgos University Hospital, Burgos, Spain

## Introduction

Tracheostomy is a procedure indicated in prolonged mechanical ventilated (MV) patients. There are controversies regarding the technique, optimal timing, its' influence on MV1 duration, hospital stay and mortality². The divergence of results in the literature justifies further research.

## Objectives

The aim of this study is to know the characteristics of patients with tracheostomy, adecuated technique, timing of realization and evolution.

## Methods

Observational study of a cohort of patients admitted to ICU during 2012, requiring elective tracheostomy (TQ), according with the actual technique protocol. Demographic variables were analysed, Apache II scale, previous pathologies, MV days, stay and mortality; globally and by groups depending on early or late TQ. In our model, patients were divided into early and late TQ, using the fourteenth day as a cutoff. Recent evidence recommends waiting 10 days to confirm the need for TQ^4^. The study was approved under the rules of the Ethics Research Commettee.

## Results

In our sample of 42 patients mean age was 61.36 years, and median Apache II 18. The most frequent indication was neurocritical patients, being percutaneous TQ in 71.5%, with 20% minor complications. Median days on VM to TQ was 14, IQR [9-17]. Both groups were comparable in age, sex and severity scale. In the early group less number of MV days and stay were significantly observed. There was no significant association between time of TQ and survival (table 1)

**Table 1:** Table 1

	Sample n = 42	Early TQ (up to 14 days of MV) n = 28	Late TQ (after 14 days of MV) n = 14	p value
Days of MV (mean,IC 95%)	26.52 (22.44-30.67)	21.37 (18.37-25.70)	35.80 (29.43-43.92)	0,000
Days of stay (mean IC 95%)	28.05 (23.85-32.24)	23.19 (19.58-27.58)	36.80 (30.35-44.73)	0,001
Survival % (Recovery/death)	64.3/35.7	74/26	46.7/53.3	0,076

## Conclusions

Elective tracheostomy is a frequent procedure used in the ICU. The percutaneous technic is the most often used with few complications according with the actual protocol. In neurocritical patients with prolonged weaning its early realization reduces cost effectively the days of MV and stay with no mortality benefit.
